# Supplementary material for: Strong and early monkeypox virus-specific immunity associated with mild disease after intradermal clade-IIb-infection in CAST/EiJ-mice
Source: Nat Commun. 2025 Feb 18;16:1729. doi: 10.1038/s41467-025-56800-2 (PMC11836108; doi:10.1038/s41467-025-56800-2)
Supplement: Supplementary file 1 — Supplementary Information [file 41467_2025_56800_MOESM1_ESM.pdf]

SUPPLEMENTAL INFORMATION

Strong and early monkeypox virus-specific immunity associated with mild disease after intradermal clade-IIb-infection in CAST/EiJ-mice

Meyer zu Natrup et al.

Supplementary Figure 1

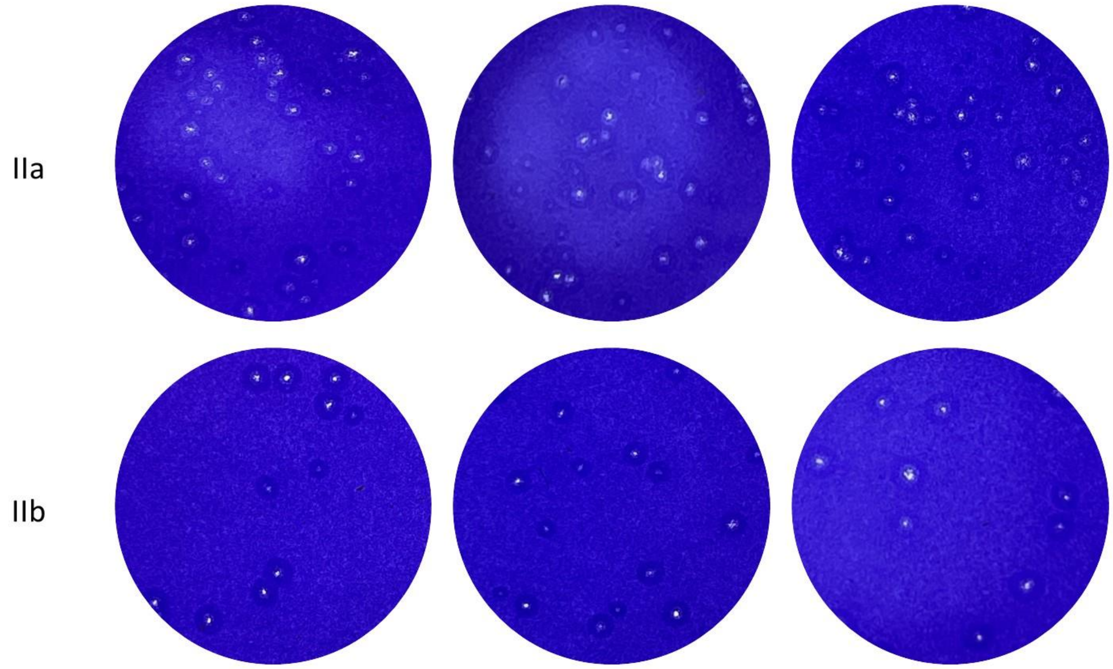

Supplementary Figure 1. Increased formation of satellite (comet) plaques after clade IIa infection. MA-104 cells were infected with ~25 PFU of MPXV clade IIa or MPXV clade IIb and were incubated for 1 h at 37°C. Unabsorbed virus was removed, and incubation continued for 48 hours without a methylcellulose overlay, followed by staining with crystal violet.

Supplementary Figure 2

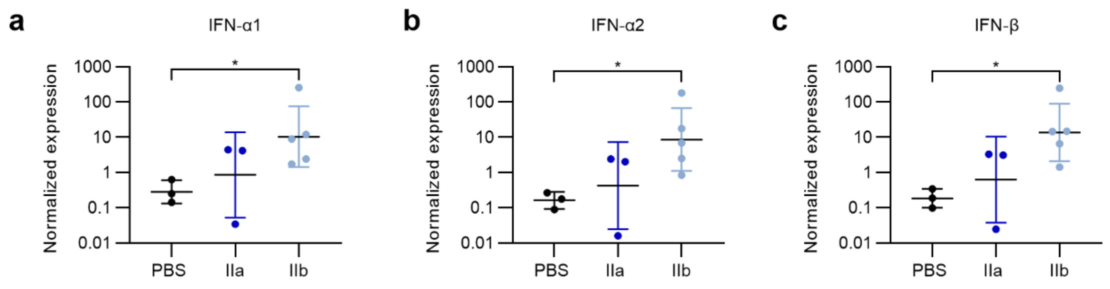

Supplementary Figure 2. **Intranasal MPXV clade IIb infection results in a stronger cytokine expression.** CAST/EiJ were intranasally infected with 2×10^5 PFU of MPXV clade IIa, clade IIb or PBS as a control. On day of sacrifice, lungs were harvested and mRNA was isolated from lung tissue to generate a cytokine profile. Cytokine levels are depicted as relative expression levels normalized against *β-actin*. Lung cytokine levels are analysed for (**a**) *IFN-α1*, (**b**) *IFN-α2* and (**c**) *IFN-β*. Error bars show the geometric mean ± geometric standard deviation (SD) (n = 3 for PBS and IIa, n = 5 for IIb). P values were determined by two-tailed Mann-Whitney test (a, b) and Kruskal-Wallis test with Dunn´s multiple comparisons test (c). *p = 0.0357 PBS versus IIb in a. *p = 0.0357 PBS versus IIb in b. *p = 0.0416 PBS versus IIb in c. Source data are provided as a Source Data file.

Supplementary Figure 3

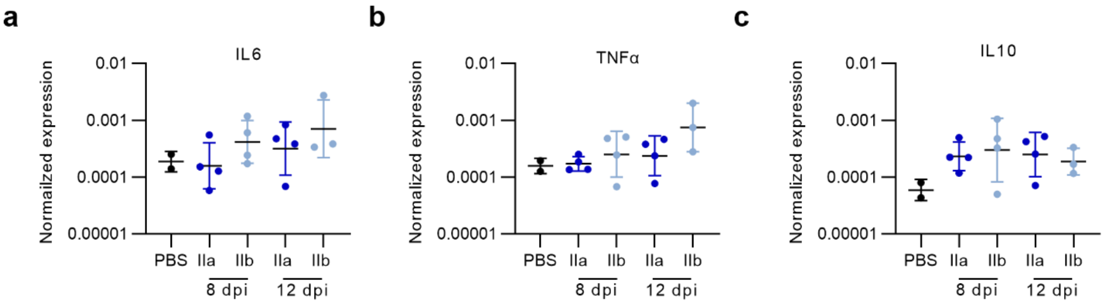

Supplementary Figure 3. Intradermal MPXV clade IIb infection results in stronger cytokine expression. CAST/EiJ were infected with 2x10^5 PFU of MPXV clade IIa, clade IIb or PBS as a control using skin tail scarification. Lungs were harvested on day of necropsy (8 dpi or 12 dpi) and mRNA was isolated from lung tissue to generate a cytokine profile, including (**a**) *IL6*, (**b**) *TNFα* and (**c**) *IL10*. Cytokine levels are depicted as relative expression levels normalized against *β-actin*. Data from mice that were euthanized prematurely due to humane endpoint assessment are excluded. Error bars show the geometric mean ± geometric standard deviation (SD) (n = 2 for PBS, n = 4 for IIa 8 dpi and IIa 12 dpi and IIb 8 dpi, n = 3 for IIb 12 dpi). Source data are provided as a Source Data file.
