# Supplementary material for: Strong and early monkeypox virus-specific immunity associated with mild disease after intradermal clade-IIb-infection in CAST/EiJ-mice
Source: Nat Commun. 2025 Feb 18;16:1729. doi: 10.1038/s41467-025-56800-2 (PMC11836108; doi:10.1038/s41467-025-56800-2)
Supplement: Supplementary file 2 — Reporting Summary [file 41467_2025_56800_MOESM2_ESM.pdf]

nature portfolio

Corresponding author(s): Asisa Volz

Last updated by author(s): Jan 21, 2025

Reporting Summary

Nature Portfolio wishes to improve the reproducibility of the work that we publish. This form provides structure for consistency and transparency in reporting. For further information on Nature Portfolio policies, see our Editorial Policies and the Editorial Policy Checklist.

Statistics

For all statistical analyses, confirm that the following items are present in the figure legend, table legend, main text, or Methods section.

n/a	Confirmed	
☐	☒	The exact sample size (*n*) for each experimental group/condition, given as a discrete number and unit of measurement
☒	☐	A statement on whether measurements were taken from distinct samples or whether the same sample was measured repeatedly
☐	☒	The statistical test(s) used AND whether they are one- or two-sided<br>*Only common tests should be described solely by name; describe more complex techniques in the Methods section.*
☒	☐	A description of all covariates tested
☐	☒	A description of any assumptions or corrections, such as tests of normality and adjustment for multiple comparisons
☐	☒	A full description of the statistical parameters including central tendency (e.g. means) or other basic estimates (e.g. regression coefficient) AND variation (e.g. standard deviation) or associated estimates of uncertainty (e.g. confidence intervals)
☐	☒	For null hypothesis testing, the test statistic (e.g. *F*, *t*, *r*) with confidence intervals, effect sizes, degrees of freedom and *P* value noted<br>*Give P values as exact values whenever suitable.*
☒	☐	For Bayesian analysis, information on the choice of priors and Markov chain Monte Carlo settings
☒	☐	For hierarchical and complex designs, identification of the appropriate level for tests and full reporting of outcomes
☒	☐	Estimates of effect sizes (e.g. Cohen's *d*, Pearson's *r*), indicating how they were calculated

Our web collection on statistics for biologists contains articles on many of the points above.

Software and code

Policy information about availability of computer code

Data collection	Microsoft Excel 2016, CTL Switchboard 2.7.2 (BioSpot Software), CFX Maestro Version 2.3 (Bio-Rad)
Data analysis	GraphPad Prism Version 9.0.0 (121), CFX Maestro Version 2.3 (Bio-Rad)

For manuscripts utilizing custom algorithms or software that are central to the research but not yet described in published literature, software must be made available to editors and reviewers. We strongly encourage code deposition in a community repository (e.g. GitHub). See the Nature Portfolio guidelines for submitting code & software for further information.

Data

Policy information about availability of data

All manuscripts must include a data availability statement. This statement should provide the following information, where applicable:

- Accession codes, unique identifiers, or web links for publicly available datasets
- A description of any restrictions on data availability
- For clinical datasets or third party data, please ensure that the statement adheres to our policy

All the data generated in this study are provided in the Source Data file. Source data are provided with this paper.

Research involving human participants, their data, or biological material

Policy information about studies with human participants or human data. See also policy information about sex, gender (identity/presentation), and sexual orientation and race, ethnicity and racism.

Reporting on sex and gender	N/A
Reporting on race, ethnicity, or other socially relevant groupings	N/A
Population characteristics	N/A
Recruitment	N/A
Ethics oversight	N/A

Note that full information on the approval of the study protocol must also be provided in the manuscript.

Field-specific reporting

Please select the one below that is the best fit for your research. If you are not sure, read the appropriate sections before making your selection.

[x] Life sciences [ ] Behavioural & social sciences [ ] Ecological, evolutionary & environmental sciences

For a reference copy of the document with all sections, see nature.com/documents/nr-reporting-summary-flat.pdf

Life sciences study design

All studies must disclose on these points even when the disclosure is negative.

Sample size	Sample sizes were not predetermined and are indicated in the figure legends. The group sizes of mice and samples were chosen based on our experience with similar studies, common practice in this field and resource availability. References: Americo, J. L. Proc Natl Acad Sci U S A 120, e2220415120, doi:10.1073/pnas.2220415120 (2023) and Volz A, J Virol. Sep;88(18):10946-57. doi: 10.1128/JVI.00945-14 (2014). In total, 41 animals were used in the study.
Data exclusions	No data were excluded.
Replication	Due to the implementation of the 3Rs principles in Germany in due of animal welfare we were not allowed to replicate animal experiments.
Randomization	Animals were randomized into different treatment groups. Samples were further randomized and serially labeled prior to use in the various assays.
Blinding	Investigators were not blinded to group allocation during animal experiment and data/sample collection due to BSL-3 safety implications. Samples were further randomized and serially labeled prior to use in the various assays and the investigators for the different assays used in the study were blinded to the identity of the samples for analysis.

Reporting for specific materials, systems and methods

We require information from authors about some types of materials, experimental systems and methods used in many studies. Here, indicate whether each material, system or method listed is relevant to your study. If you are not sure if a list item applies to your research, read the appropriate section before selecting a response.

Materials & experimental systems

n/a	Involved in the study	
[ ]	[x]	Antibodies
[ ]	[x]	Eukaryotic cell lines
[x]	[ ]	Palaeontology and archaeology
[ ]	[x]	Animals and other organisms
[x]	[ ]	Clinical data
[x]	[ ]	Dual use research of concern
[x]	[ ]	Plants

Methods

n/a	Involved in the study	
[x]	[ ]	ChIP-seq
[x]	[ ]	Flow cytometry
[x]	[ ]	MRI-based neuroimaging

Antibodies

Antibodies used	The following antibodies were used for immunostaining:

	Primary antibody: Vaccinia Virus (Lister Strain) Rabbit Polyclonal Antibody, Origene, cat.: BP1076, lot: 5G18723, polyclonal, 1:2000 diluted<br>Secondary antibody: Peroxidase-conjugated AffiniPure(TM) Goat Anti-Rabbit IgG (H+L) (min X Hu,Ms,Rat Sr Prot), Biozol, cat.: JIM-111-035-144, lot: 166835, polyclonal, 1:5000 diluted
Validation	Vaccinia Virus (Lister Strain) Rabbit Polyclonal Antibody, OriGene, BP1076: https://www.origene.com/catalog/antibodies/primary-antibodies/bp1076/vaccinia-virus-lister-strain-rabbit-polyclonal-antibody<br>secondary HRP-labeled goat anti-rabbit antibody (Biozol, JIM-111-035-144): https://www.biozol.de/de/product/jim-111-035-144<br>In addition, antibodies were titrated using well-characterized MVA-stocks and have been extensively used in previous studies: Tscherne et al. PNAS 2021 doi: 10.1073/pnas.2026207118, Meyer zu Natrup et al. JCI 2022 doi:10.1172/JCI159895.

Eukaryotic cell lines

Policy information about cell lines and Sex and Gender in Research

Cell line source(s)	All cell lines are from ATCC. MA-104 (CRL-2378.1) is an epithelial cell from the kidney of an African green monkey. Vero E6 (CRL-1586) was isolated from the kidney of an African green monkey.
Authentication	Cell lines were not authenticated.
Mycoplasma contamination	All cell lines are regularly tested for mycoplasma. For this purpose, supernatant is taken from which DNA is isolated. Mycoplasma DNA is analysed using PCR methodology. No specific mycoplasma DNA was detected in this case. All cell lines used within this study were confirmed negative.
Commonly misidentified lines<br>(See ICLAC register)	We used MA-104 cells in our study. This cell line is used for the propagation of MPXV.

Animals and other research organisms

Policy information about studies involving animals; ARRIVE guidelines recommended for reporting animal research, and Sex and Gender in Research

Laboratory animals	Mouse, CAST/EiJ, 12 - 20 weeks, n = 61, originating from Jackson Laboratories
Wild animals	The study did not involve wild animals.
Reporting on sex	Both female and male individuals were used in this study. The obtained results were not categorised by sex because no sex differences were identified in CAST/EIJ mice.
Field-collected samples	The study did not involve samples collected from the field.
Ethics oversight	The experiments were approved by the Niedersächsisches Landesamt für Verbraucherschutz und Lebensmittelsicherheit (LAVES) in Lower Saxony, Germany (33.19-42502-04-22-00207). The protocol was approved by the Commission according to section 15 of the German Animal Welfare Act and the Commission for Research Ethics of the University.

Note that full information on the approval of the study protocol must also be provided in the manuscript.

Plants

Seed stocks	N/A
Novel plant genotypes	N/A
Authentication	N/A
